# Supplementary material for: Shifts in isoform usage underlie transcriptional differences in regulatory T cells in type 1 diabetes
Source: Commun Biol. 2023 Sep 27;6:988. doi: 10.1038/s42003-023-05327-7 (PMC10533491; doi:10.1038/s42003-023-05327-7)
Supplement: Supplementary file 2 — Supplementary Information [file 42003_2023_5327_MOESM2_ESM.pdf]

Supplementary Information

Supplementary Table 1. Study sample demographics 2

Supplementary Table 2. Antibody panels for flow cytometry 3

Supplementary Note 1. Summary of unannotated events in differentially spliced genes 4

Supplementary Figure 1. Distribution of normalized transcripts-per-million 5

Supplementary Figure 2. Gene and protein expression of *CXCR3*, *IL6*/CD126, and *STAMBPL1andFAS*/CD95 6

Supplementary Figure 3. Summary of genes with unannotated splicing events 8

Supplementary Figure 4. Purity of memory $CD4^+$/$CD25^+$ populations 9

Supplementary Table 1. Study sample demographics

	All subjects		memory $CD4^+/CD25^+$ regulatory T cells (total)		memory $CD4^+/CD25^-$ T cells (total)		memory $CD4^+/CD25^+$ regulatory T cells (analyzed)		memory $CD4^+/CD25^-$ T cells (analyzed)	
	**T1D cases**	**Controls**	**T1D cases**	**Controls**	**T1D cases**	**Controls**	**T1D cases**	**Controls**	**T1D cases**	**Controls**
Total subjects	81	77	55	44	67	66	49	35	53	52
Female / Male	40 / 41	39 / 38	30 / 25	21 / 23	28 / 39	32 / 34	27 / 22	16/ 19	20 / 33	26 / 26
Mean age ± SD	32.5 ± 7.9	32.8 ± 7.5	32.6 ± 7.5	34.3 ± 8.0	32.8 ± 8.2	32.6 ± 7.7	33.2 ± 7.5	34.5 ± 8.5	33.3 ± 8.5	32.8 ± 8.0
Mean age at diagnosis ± SD	19.2 ± 9.9	n/a	18.8 ± 9.2	n/a	19.2 ± 10.1	n/a	18.9 ± 9.2	n/a	20.0 ± 10.3	n/a
Mean disease duration ± SD	13.6 ± 10.5	n/a	14.3 ± 10.6	n/a	13.9 ± 10.4	n/a	14.8 ± 10.9	n/a	13.6 ± 10.6	n/a

Supplementary Table 2: Antibody panels for flow cytometry data.

Panel	Target#	Clone	Format	Supplier	Lot number
Panel 1 (Tdiff)^: Figure 2E, Supplemental Figure 2	CD25	M-A251	FITC	BD	4181913
	CD4	SK3	BV786	BD	4037603
	CD3	SK7(leu-4)	APC-H7	BD	4010832
	CXCR3 (CD183)	LS177-1C6	PE-Cy5	BD	4148516
	FOXP3	259D/C7	AF647	BD	3325709
Panel 2 (Tact)^: Supplemental Figure 2	CD127	HIL-7R-M21	FITC	BD	4035685
	CD3	UCHT1	PerCP Cy5.5	BD	4114896
	CD95	DX2	APC	BD	3099509
	CD126	MS	PE	BD	3151929
	CD25	M-A251	PE-Cy7	BD	3301965
Purity check^:	CD4	RPA-T4	PerCP-Cy5.5	BL	300350
	CD45RO	UCHL1	APC	BD	559865
	CD25	4E3	PE	Miltenyi	130091

All reagents were validated for specificity and purity by Becton Dickinson (BD).

^ Treg gating for Panel 1: FWD/SSC, singlet, live dead, CD3, CD25xFOXP3 (Treg), MFI of FOXP3 and CXCR3 on $CD25^{+}FOXP3^{+}$ Treg. Treg gating for Panel 2: FWD/SSC, singlet, live dead, CD3, CD25xCD127 (Treg), MFI of CD95 and CD126 on $CD25^{+}CD127^{-}$ Treg. Purity check: FWD/SSC, singlet, live dead, CD4xCD45RO, CD25.

Supplementary Note 1. Summary of unannotated events in differentially spliced genes

Given the overrepresentation of splicing or RNA-binding genes and the abundance of intron retention in differentially spliced genes in Tregs (Supplementary Data 1), we examined whether novel combinations of existing donor/acceptor sites (unannotated junctions) and/or possible intron read-through (exon-intron border junctions) were more likely to be detected in differentially spliced genes or genes with differential exon fragment usage. Most unannotated transcriptional events (collectively unannotated junctions and exon-intron border junctions) were detected in both T1D cases and controls in memory $CD4^+/CD25^-$ T cells (86% of genes with unannotated junctions and 90% of genes with border junctions common to both cases and controls; Supplementary Figure 3a). In Tregs, unannotated events were more frequently specific to either T1D cases or controls (68% of genes with unannotated junctions and 83% of genes with border junctions common to both cases and controls; Supplementary Figure 3b). Limiting the analyses to the multi-transcript genes represented in the reduced transcriptome for Tregs, unannotated junctions were more frequently detected in differentially spliced genes than in non- differentially spliced genes (112/402 differentially spliced genes; 429/2,132 non-differentially spliced genes; P=0.0005). In addition, twice as many genes with differential exon fragment usage expressed unannotated junctions compared with genes not displaying evidence of differential exon fragment usage in Tregs (226/1,112 genes with differential exon fragment usage (20%), 1,155/11,058 genes without differential exon fragment usage genes (10%); P<0.0001), and three times as many genes with unannotated events were exclusive to either T1D cases or controls (108/1,112 genes genes with differential exon fragment usage and differentially-detected unannotated junctions (10%); 362/11,058 genes with only differentially-detected unannotated junctions (3%); P<0.0001). Overall, a higher fraction of genes with unannotated events were exclusive to T1D cases in Tregs.

Splicing factor genes were also more likely to express unannotated events than other genes in Tregs (13/55 splicing factor genes (24%); 1,368/12,115 other genes (11%); P=0.004).

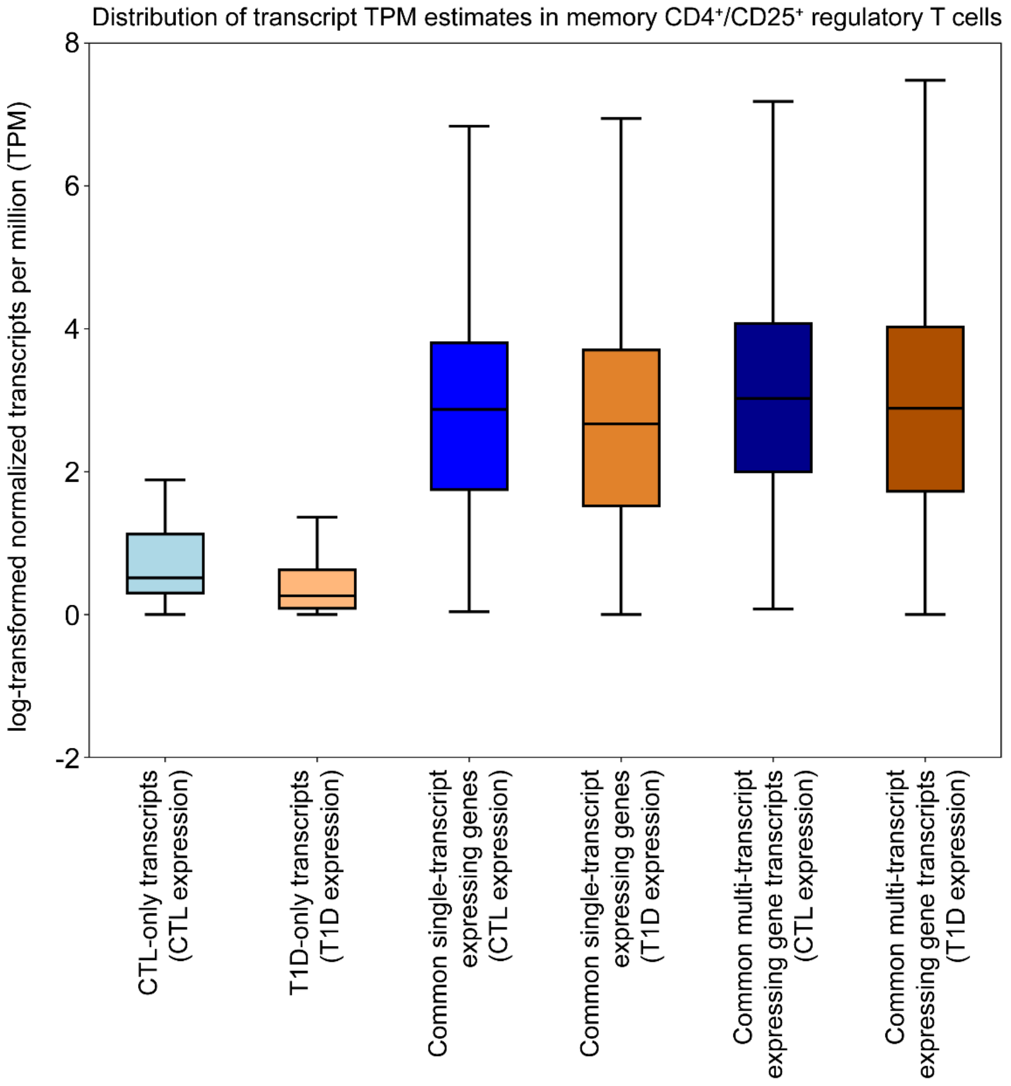

Supplementary Figure 1. Transcript expression summary. Distribution of normalized transcripts-per-million estimates of EA-reduced transcripts expressed in T1D cases and/or unaffected controls by their detection and transcriptional complexity. N = 31 transcripts with only CTL expression (median TPM = 0.67, interquartile range = 0.35 – 2.08); 90 transcripts with

only T1D expression (median TPM = 0.30, interquartile range = 0.09 – 0.87); 5,909 transcripts from single-transcript-expressing genes expressed in both CTL and T1D (CTL: median TPM = 16.65, interquartile range = 4.74 – 43.76; T1D: median TPM = 13.43, interquartile range = 3.58 – 39.56); 8,238 transcripts from multiple-transcript-expression genes expressed in both CTL and T1D (CTL: median TPM = 19.62, interquartile range = 6.39 – 57.86; T1D: median TPM = 16.92, interquartile range = 4.61 – 55.15). Data for this plot are available in Supplementary Data 7. Upper error bars are calculated as the third quartile + 1.5 × interquartile range, lower error bars are calculated as first quartile - 1.5 × interquartile range.

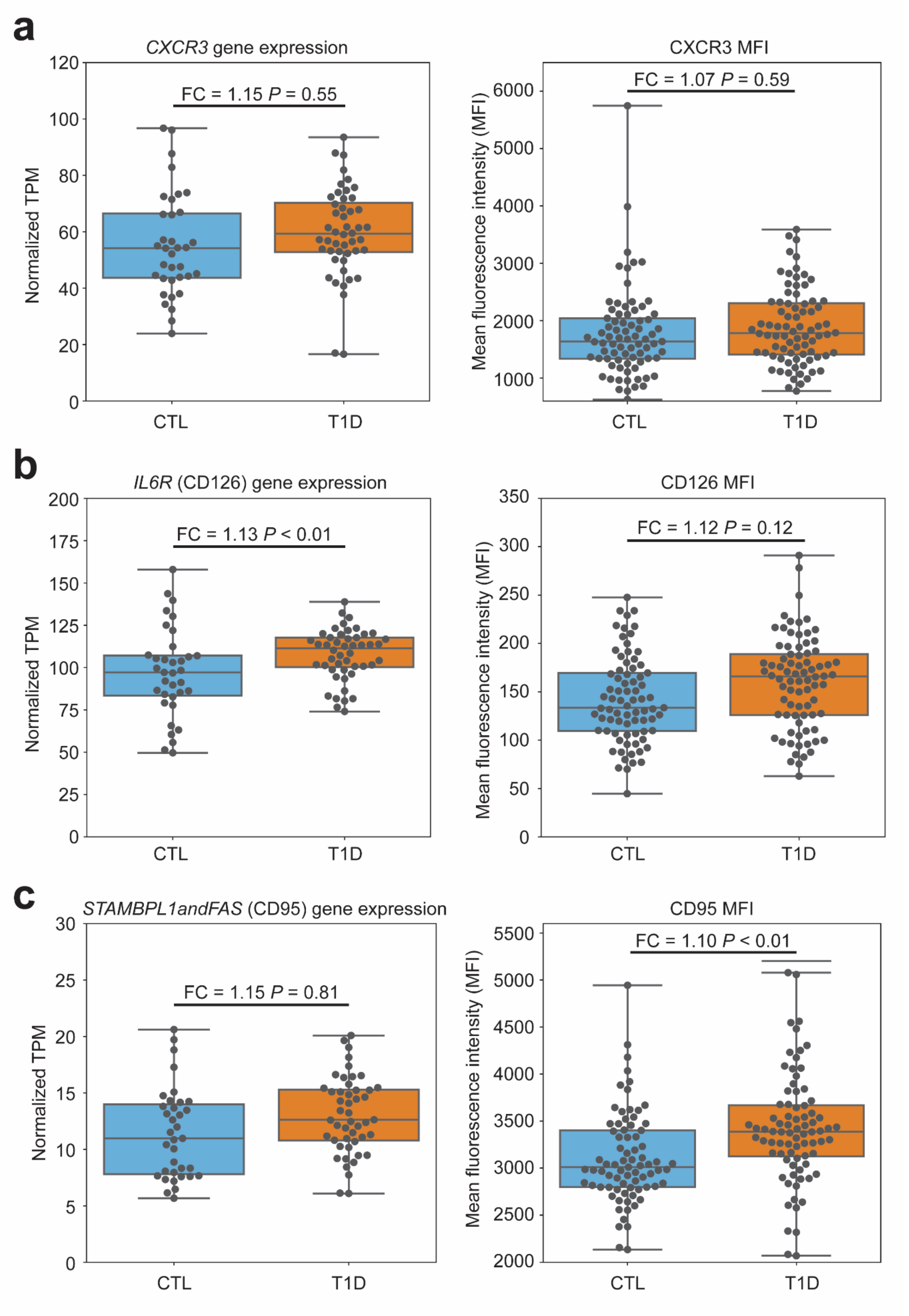
a
CXCR3 gene expression
FC = 1.15 P = 0.55
Normalized TPM
CTL
T1D
CXCR3 MFI
FC = 1.07 P = 0.59
Mean fluorescence intensity (MFI)
CTL
T1D
b
IL6R (CD126) gene expression
FC = 1.13 P < 0.01
Normalized TPM
CTL
T1D
CD126 MFI
FC = 1.12 P = 0.12
Mean fluorescence intensity (MFI)
CTL
T1D
c
STAMBPL1andFAS (CD95) gene expression
FC = 1.15 P = 0.81
Normalized TPM
CTL
T1D
CD95 MFI
FC = 1.10 P < 0.01
Mean fluorescence intensity (MFI)
CTL
T1D

Supplementary Figure 2. Gene and protein expression of immune cell markers in Tregs. Distribution of normalized transcripts per million (TPM) for the *FOXP3* gene mean fluorescent intensity (MFI) of the FOXP3 protein in memory $CD4^+/CD25^+$ regulatory T cells for **a** the *CXCR3* gene (CTL: N = 35, median TPM = 54.16, interquartile range = 43.66 – 66.51; T1D: N = 48, median TPM = 59.27, interquartile range = 52.82 – 70.27) and its protein (CTL: N = 76, median MFI = 3,007.46, interquartile range = 2,794.94 – 3,398.66; T1D: N = 79, median MFI = 3,385.08, interquartile range = 3,121.87 – 3,666.10); **b** the *IL6R* gene (CTL: N = 35, median TPM = 97.16, interquartile range = 83.48 – 107.17; T1D: N = 48, median TPM = 111.43, interquartile range = 100.21 – 117.70) and its protein CD126 (CTL: N = 76, median MFI = 133.50, interquartile range = 109.47 – 169.49; T1D: N = 79, median MFI = 165.84, interquartile range = 125.89 – 188.98); and **c** the *STAMBPL1andFAS* Aceview locus containing the *FAS* gene (CTL: N = 35, median TPM = 10.98, interquartile range = 7.81 – 13.99; T1D: N = 48, median TPM = 12.61, interquartile range = 10.79 – 15.29) and its corresponding protein CD95 (CTL: N = 76, median MFI = 1,638.06, interquartile range = 1,337.58 – 2,043.68; T1D: N = 79, median MFI = 1,783.66, interquartile range = 1,409.04 – 2,304.50). FC = fold change calculated as the mean type 1 diabetics (T1D) value divided by the mean control (CTL) value. Data for boxplots are available in Supplementary Data 3 (panels **a**, **c**, and **e**) and Supplementary Data 4 (panels **b**, **d**, and **f**). Upper error bars are calculated as the third quartile + 1.5 × interquartile range, lower error bars are calculated as first quartile - 1.5 × interquartile range.

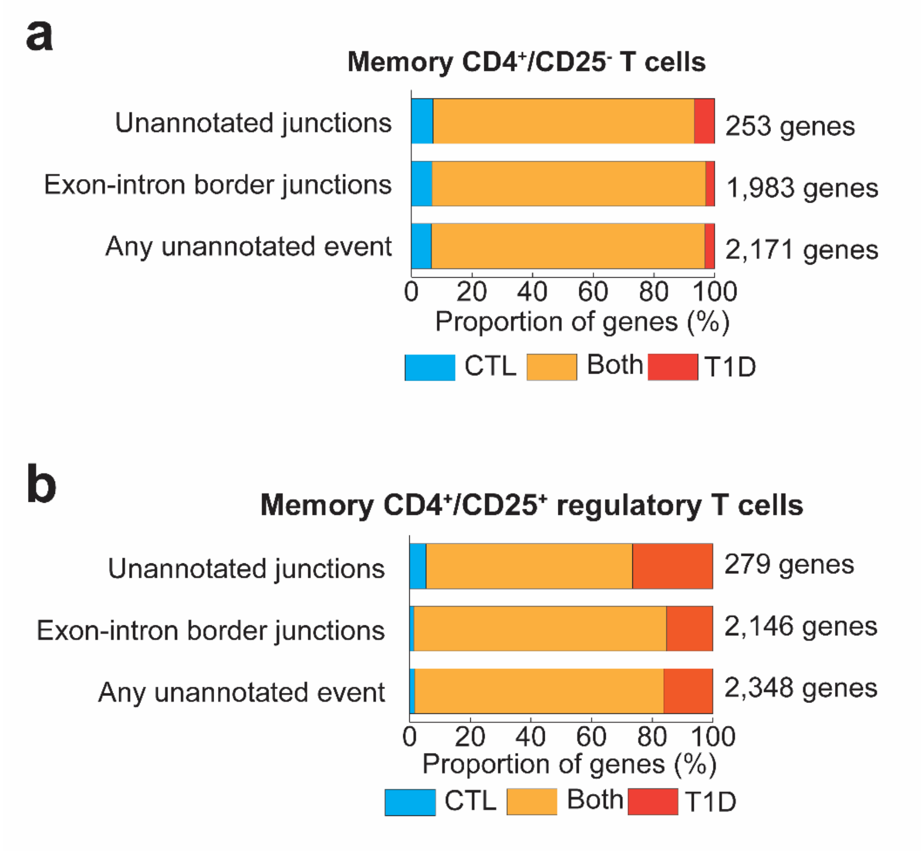

Supplementary Figure 3. Detection of unannotated splicing events. Summary of genes with unannotated splicing events (unannotated junctions, exon-intron border junctions, or either) for **a** memory CD4^{+}/CD25^{-} T cells and **b** memory CD4^{+}/CD25^{+} regulatory T cells. Data for these plots are available in Supplementary Data 8.

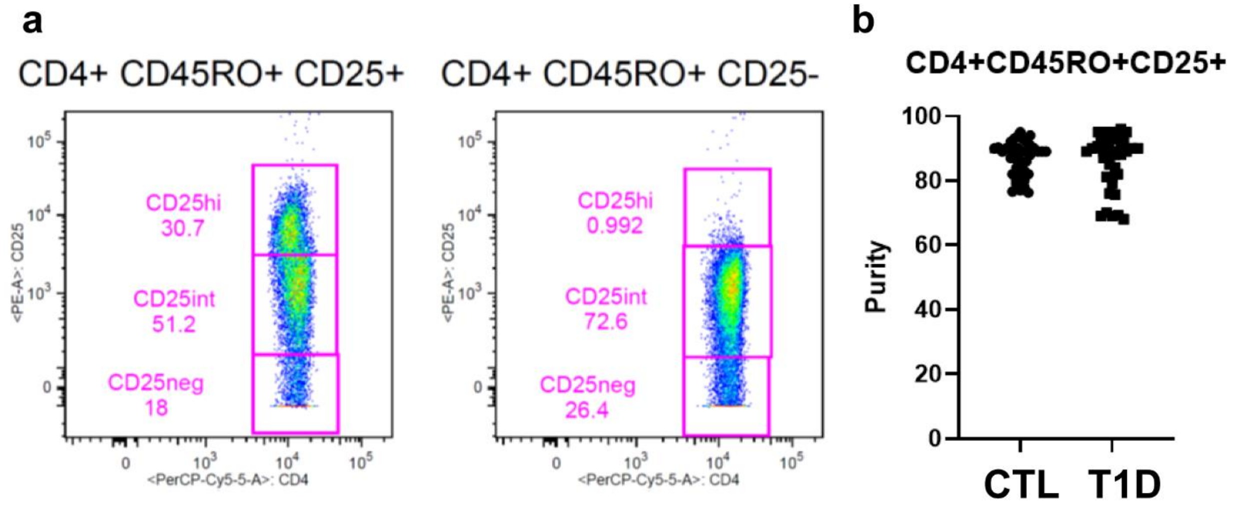

Supplementary Figure 4. Purity of memory CD4⁺/CD25⁺ populations. **a** Representative gating of populations to determine purity of CD4⁺/CD25⁺ and CD4⁺/CD25⁻ cells. **b** Purity was determined approximately weekly on a subset of samples by comparing CD25^{hi} in the input CD4^{+}CD45RO^{+} population and the CD45RO^{+}/CD25^{-} negative fraction by flow cytometry. Controls (CTL) and type 1 diabetic (T1D) purities did not differ significantly. The purity panel was acquired on a BD Canto using Diva software and analyzed using FloJo software version 7.7 (Ashland, OR).
