# Supplementary material for: Shifts in isoform usage underlie transcriptional differences in regulatory T cells in type 1 diabetes
Source: Commun Biol. 2023 Sep 27;6:988. doi: 10.1038/s42003-023-05327-7 (PMC10533491; doi:10.1038/s42003-023-05327-7)
Supplement: Supplementary file 3 — Description of Additional Supplementary Files [file 42003_2023_5327_MOESM3_ESM.pdf]

Description of Additional Supplementary Files

File name: Supplementary Data 1
Description: Differentially-spliced genes in memory $CD4^+$/$CD25^+$ regulatory T cells with percent spliced in of major splicing differences between isoforms.

File name: Supplementary Data 2
Description: Data for Figure 2a. This includes the feature counts for Figure 2a, detection summaries of exon fragments, junctions, and transcripts.

File name: Supplementary Data 3
Description: Data for Figures 2d (*FOXP3* gene expression) and Supplementary Figures 2a, 2c, and 2e (*CXCR3*, *IL6*, and *STAMBPL1andFAS* gene expression).

File name: Supplementary Data 4
Description: Data for Figures 2e (FOXP3 mean fluorescence intensity) and Supplementary Figures 2b, 2d, and 2f (CXCR3, CD126, and CD95 mean fluorescence intensities).

File name: Supplementary Data 5
Description: Median and interquartile ranges for Figures 4b (*FOXP3* isoform percentage), 4c (*FOXP3* transcript and total gene expression), 5b (*TRA2B* isoform percentage), and 5c (*TRA2B* transcript and total gene expression).

File name: Supplementary Data 6
Description: Count data for Figures 4b (*FOXP3* isoform percentage) and 4c (*FOXP3* transcript and total gene expression).

File name: Supplementary Data 7
Description: Count data for Figures 5b (*TRA2B* isoform percentage) and 5c (*TRA2B* transcript and total gene expression).

File name: Supplementary Data 8
Description: Data for Supplementary Figure 1: mean normalized expression of transcripts detected in memory $CD4^+$/$CD25^+$ regulatory T cells from controls and T1D cases.

File name: Supplementary Data 9
Description: Data for Supplementary Figure 3: summary of unannotated splicing events detected in memory $CD4^+$/$CD25^+$ regulatory T cells and memory $CD4^+$/$CD25^-$ T cells.
