# Supplementary material for: Shifts in isoform usage underlie transcriptional differences in regulatory T cells in type 1 diabetes
Source: Commun Biol. 2023 Sep 27;6:988. doi: 10.1038/s42003-023-05327-7 (PMC10533491; doi:10.1038/s42003-023-05327-7)
Supplement: Supplementary file 13 — Reporting Summary [file 42003_2023_5327_MOESM13_ESM.pdf]

nature portfolio

Corresponding author(s): Pat Concannon

Last updated by author(s): Aug 15, 2023

Reporting Summary

Nature Portfolio wishes to improve the reproducibility of the work that we publish. This form provides structure for consistency and transparency in reporting. For further information on Nature Portfolio policies, see our Editorial Policies and the Editorial Policy Checklist.

Statistics

For all statistical analyses, confirm that the following items are present in the figure legend, table legend, main text, or Methods section.

n/a	Confirmed	
☐	☒	The exact sample size (*n*) for each experimental group/condition, given as a discrete number and unit of measurement
☐	☒	A statement on whether measurements were taken from distinct samples or whether the same sample was measured repeatedly
☐	☒	The statistical test(s) used AND whether they are one- or two-sided<br>*Only common tests should be described solely by name; describe more complex techniques in the Methods section.*
☐	☒	A description of all covariates tested
☐	☒	A description of any assumptions or corrections, such as tests of normality and adjustment for multiple comparisons
☐	☒	A full description of the statistical parameters including central tendency (e.g. means) or other basic estimates (e.g. regression coefficient) AND variation (e.g. standard deviation) or associated estimates of uncertainty (e.g. confidence intervals)
☐	☒	For null hypothesis testing, the test statistic (e.g. *F*, *t*, *r*) with confidence intervals, effect sizes, degrees of freedom and *P* value noted<br>*Give P values as exact values whenever suitable.*
☒	☐	For Bayesian analysis, information on the choice of priors and Markov chain Monte Carlo settings
☒	☐	For hierarchical and complex designs, identification of the appropriate level for tests and full reporting of outcomes
☐	☒	Estimates of effect sizes (e.g. Cohen's *d*, Pearson's *r*), indicating how they were calculated

Our web collection on statistics for biologists contains articles on many of the points above.

Software and code

Policy information about availability of computer code

Data collection	Sample RNA were sequenced on an Illumina HiSeq 2000 instrument and saved as BCL files. The program bcl2fastq was used to demultiplex reads into individual samples as FASTQ files.
Data analysis	Transcript estimates were calculated from RNA-seq FASTQ files using the RSEM algorithm; transcriptional event estimates were calculated using Event Analysis python scripts (https://github.com/McIntyre-Lab/events). All data analyses were conducted in SAS v9.4 and JMP Genomics v9. Custom SAS programs used in the analysis of the data are available (https://github.com/jrbnewman/T1D_treg_splicing/tree/master). All code pertaining to the analysis presented in this study has been deposited as a Zenodo archive (doi:10.5281/zenodo.8226066), and can additionally be found at https://github.com/jrbnewman/T1D_treg_splicing/tree/master

For manuscripts utilizing custom algorithms or software that are central to the research but not yet described in published literature, software must be made available to editors and reviewers. We strongly encourage code deposition in a community repository (e.g. GitHub). See the Nature Portfolio guidelines for submitting code & software for further information.

Data

Policy information about availability of data

All manuscripts must include a data availability statement. This statement should provide the following information, where applicable:

- Accession codes, unique identifiers, or web links for publicly available datasets
- A description of any restrictions on data availability
- For clinical datasets or third party data, please ensure that the statement adheres to our policy

The sequencing data from this study have been submitted to the NCBI Gene Expression Omnibus (GEO; https://www.ncbi.nlm.nih.gov/geo) under accession number GSE237218. Source data are provided in Supplementary Data 1-8 available on FigShare (doi:10.6084/m9.figshare.22789763).

Human research participants

Policy information about studies involving human research participants and Sex and Gender in Research.

Reporting on sex and gender	Chromosome X and Y SNP genotype calls were used to confirm subject sex. In addition, the expression of the genes TISX and XIST (chromosome X genes involved in X-inactivation) and EIF1AY (chromosome Y) was also examined. The ratio of EIF1AY to TISX/XIST expression was calculated, where a high EIF1AY:XIST ratio indicated a male subject and a low or zero ratio indicated a female subject. The initial study population from which coded samples were provided included equal numbers of male and female subjects. Final numbers studied, after sample exclusions for technical or quality control reasons, are listed in Supplementary Table 2. No sex-specific analyses were performed as results are expected to apply to both sexes.
Population characteristics	This study reports the results of analyses of coded samples. No subjects were recruited for the study. Information regarding population characteristics provided with the coded samples is provided in Supplementary Table 2.
Recruitment	No human subjects were recruited for the studies described. Only pre-existing samples, identified with codes that provided no information regarding the subjects from whom they were derived, were used.
Ethics oversight	All samples were collected under protocols approved by the Benaroya Research Institute IRB (IRB-07109), with written informed consent obtained from all study participants.

Note that full information on the approval of the study protocol must also be provided in the manuscript.

Field-specific reporting

Please select the one below that is the best fit for your research. If you are not sure, read the appropriate sections before making your selection.

☒ Life sciences ☐ Behavioural & social sciences ☐ Ecological, evolutionary & environmental sciences

For a reference copy of the document with all sections, see nature.com/documents/nr-reporting-summary-flat.pdf

Life sciences study design

All studies must disclose on these points even when the disclosure is negative.

Sample size	Final sample size was dependent on sample availability in the repository from which samples were drawn, as well as the impact of exclusions for technical (e.g. insufficient RNA for analysis) or quality control reasons.
Data exclusions	As described in the Quality Control heading in the Supplementary Materials section, samples were excluded from analysis if they failed to cluster with the appropriate cell type in a principal components analysis, were related to another study subject in a kinship analysis, or if genotypes determined from RNA-seq data were discordant with separately determined genotypes derived from DNA analysis. These exclusion criteria represent standard quality control parameters and were pre-established.
Replication	In the revised manuscript, we have included protein data, obtained via flow cytometry, for a limited number of genes as a validation requested by the reviewers.
Randomization	Our report is a retrospective study of cases and controls. No assignment to treatment groups was performed.
Blinding	The study was performed using coded cell samples. After RNA extraction, samples were recoded and subsequent library construction and RNA-seq was performed by investigators blinded with regard to all information related to the samples. DNA genotypes used for quality control testing were determined by a separate laboratory using separately and uniquely coded samples. Investigators carrying out the genotyping had no access to information related to the samples being processed and were not involved in the RNA-seq studies performed.

Reporting for specific materials, systems and methods

We require information from authors about some types of materials, experimental systems and methods used in many studies. Here, indicate whether each material, system or method listed is relevant to your study. If you are not sure if a list item applies to your research, read the appropriate section before selecting a response.

Materials & experimental systems

n/a	Involved in the study
☐	☒ Antibodies
☒	☐ Eukaryotic cell lines
☒	☐ Palaeontology and archaeology
☒	☐ Animals and other organisms
☒	☐ Clinical data
☒	☐ Dual use research of concern

Methods

n/a	Involved in the study
☒	☐ ChIP-seq
☐	☒ Flow cytometry
☒	☐ MRI-based neuroimaging

Antibodies

Antibodies used	A table listing the commercial sources of all antibodies used has been added to the Supplementary data section as Supplementary Table 2.
Validation	Vendor's validation is noted in the footnote to Supplementary Table 2.

Flow Cytometry

Plots

Confirm that:

☒ The axis labels state the marker and fluorochrome used (e.g. CD4-FITC).

☒ The axis scales are clearly visible. Include numbers along axes only for bottom left plot of group (a 'group' is an analysis of identical markers).

☒ All plots are contour plots with outliers or pseudocolor plots.

☒ A numerical value for number of cells or percentage (with statistics) is provided.

Methodology

Sample preparation	Samples were prepared from freshly drawn whole blood. PBMCs were isolated and then positively selected on CD19 beads. The flowthrough fraction was then positively selected on CD8 beads. One half of the CD8- cells were purified on CD4 memory beads. These cells were then fractionated on CD25 beads and the resulting two populations were used for the analyses described in the current manuscript.
Instrument	The following statement appears in the Methods section under the "Immune Marker Measurements" heading: " Data were collected on a BD Fortessa cytometer using Diva software and analyzed with FlowJo software (version 7.2; TreeStar, Ashland, OR). Invitrogen 8 peak beads were used to normalize flow cytometry settings between experiments by adjusting voltage settings to reach a standard MFI.
Software	Software is cited as indicated above.
Cell population abundance	Abundance and purity of post-sort cell populations is address in Supplementary Figure 4 in the revised manuscript. This includes representative FACS plots and purities determined on a weekly basis throughout sample collection.
Gating strategy	Gating strategies for different panels are described in the footnote to Supplementary Table 2

☒ Tick this box to confirm that a figure exemplifying the gating strategy is provided in the Supplementary Information.
